# Occupational injury history and universal precautions awareness: a survey in Kabul hospital staff

**DOI:** 10.1186/1471-2334-10-19

**Published:** 2010-01-30

**Authors:** Ahmad Shah Salehi, Paul Garner

**Affiliations:** 1 Ministry of Public Health, Kabul, Afghanistan; 2 International Health Group, Liverpool School of Tropical Medicine, Liverpool, UK

## Abstract

**Background:**

Health staff in Afghanistan may be at high risk of needle stick injury and occupational infection with blood borne pathogens, but we have not found any published or unpublished data.

**Methods:**

Our aim was to measure the percentage of healthcare staff reporting sharps injuries in the preceding 12 months, and to explore what they knew about universal precautions. In five randomly selected government hospitals in Kabul a total of 950 staff participated in the study. Data were analyzed with Epi Info 3.

**Results:**

Seventy three percent of staff (72.6%, 491/676) reported sharps injury in the preceding 12 months, with remarkably similar levels between hospitals and staff cadres in the 676 (71.1%) people responding. Most at risk were gynaecologist/obstetricians (96.1%) followed by surgeons (91.1%), nurses (80.2%), dentists (75.4%), midwives (62.0%), technicians (50.0%), and internist/paediatricians (47.5%). Of the injuries reported, the commonest were from hollow-bore needles (46.3%, n = 361/780), usually during recapping. Almost a quarter (27.9%) of respondents had not been vaccinated against hepatitis B. Basic knowledge about universal precautions were found insufficient across all hospitals and cadres.

**Conclusion:**

Occupational health policies for universal precautions need to be implemented in Afghani hospitals. Staff vaccination against hepatitis B is recommended.

## Background

"Universal precautions" aim to prevent transmission of human Immunodeficiency virus (HIV), hepatitis B (HBV), and other blood borne pathogens. The objective is to assume patients are infected with blood-borne pathogens, and ensuring health staff minimise the risk of exposure to infected body fluids [1]. These measures are important, as it is estimated that the attributable fractions for percutaneous occupational exposure are 37% for hepatitis B, 39% for hepatitis C and 4.4% for HIV [2]. Hepatitis B is particularly infectious, with the risk of transmission of HBV from needlestick or other sharps injuries to health care workers ranging from 6% to 30% [3].

In Afghanistan, we do not know the population prevalence of HIV, hepatitis B virus, and hepatitis C virus. It could be high: HCV is common in injecting drug users (36.6%) [4]; regional conflict in the last two decades has meant approximately eight million Afghan migrated to neighbouring countries, particularly Iran and Pakistan, where HIV is relatively common among injecting drug users [5]. With 5.7 million Afghan returning home during 2002-2006 the risk of an HIV epidemic is high [6]. In addition, illiteracy, poverty, and subjugation of women combined with political and social instability are likely to fuel an HIV epidemic in the country [7]. Under these circumstances, there is likely to be a significant risk of transmission of blood borne infections to health staff.

In 2006, the Afghan Ministry of Public Health developed a national infection prevention strategy. The goal was to improve the quality of health care, and to limit the spread of infections in patients, healthcare workers, and the community. One component of the programme was to help health staff adopt and comply with internationally accepted standards of infection prevention practices. At the same time, the Ministry developed an infection prevention guideline, which provided infection prevention standards, and details about appropriate infection prevention practices in health facilities and mobile outreach services. However, there was no clear implementation plan.

In this survey, we aimed to measure the proportion of health staff reporting sharps injuries in the last 12 months in Kabul hospitals, and explore their understanding of standard precautions.

## Methods

In Kabul city, there are 10 national public hospitals, adult [5], maternity [2], paediatric [2] and dental [1] hospitals. Using the Ministry of Public Health list, we selected at random two general adult hospitals, one maternity hospital, and one paediatric hospital. In addition, the dental hospital was added to the list.

We adapted standard questionnaires from other studies [8-10], and added a section of 'belief', adapted from a study in a Taiwan hospital [11]. At each hospital, staff completed the questionnaire which asked about socio-demographic data, occurrence of sharps injuries in the past 12 months, their beliefs about safety, and knowledge of universal precautions.

The investigator met with hospital directors to explain the purpose of the study. At each hospital, we obtained a full list of staff currently working there; the investigator briefed all staff at their daily routine morning meeting. Anonymity and confidentiality was assured, and the right to refuse participation. After a question and answer session, information sheets and questionnaires were distributed to staff. Most of the questionnaires were returned on the spot.

### Ethical approval

We obtained ethical approval from the Research Ethics Committee of the Liverpool School of Tropical Medicine; and the Institutional Review Board of the Ministry of Public Health of Afghanistan.

### Data analysis

The collected data were imported to Epi info version 3.4.3 for analysis. We examined responses between hospitals and then combined them as they did not show large differences. Basic statistics were calculated. Differences in sharps injuries events by categories of health care workers and hospitals were quantified.

## Results

We distributed 950 questionnaires to named staff, with 676 responding (71.1%). The response rate was similar between hospitals, ranging from 68.2% to 73.3%. Respondents were nurses (27.7% n = 187), Internists/paediatricians (17.8%, n = 187), surgeons (16.4% n = 111)), midwives (14.8%, n = 100), dentists (10.2% n = 60), gynaecologist/obstetricians (7.5%, n = 51) and technicians (5.6%, n = 38); with 37.5% female (n = 254); and overall, 58.1% had 3 or more years working experience.

Sharps injuries in the past 12 months were reported in 72.6% overall (491/676); and for direct blood and body fluids contact, it was 68.0% (n = 460/676). Multiple injuries were common, with 34% [167] reported more than 3 injuries in the past 12 months. Rates ranged from 96.1% in gynaecologist/obstetricians to 47.5% in Internists/paediatricians (table 1). Injuries were less commonly reported in health staff aged 50 and above, but apart from this, rate of needlestick and sharps injuries among hospitals, gender, ethnicity, marital status, and vaccination status were similar (table 1).

**Table 1:** Needlestick injury numbers and percentage within respondent categories (n = 676)

Variables	Injury n/N (%)
**Hospital**	
Hospital A	104/130 [80]
Hospital B	130/178 [73]
Hospital C	82/110 (74.5)
Hospital D	120/183 (65.6)
Hospital E	55/75 (73.3)
TOTAL	491
**Occupation**	
Gynaecologist/Obstetrician	49/51 (96.1)
Surgeon	102/111 (91.9)
Nurse	150/187 (80.2)
Dentist	52/69 (75.4)
Midwife	62/100 (62.0)
Technician	19/38 (50.0)
Internist/paediatrician	57/120 (47.5)
**Gender**	
Female	194/254(76.4)
Male	297/422(70.4)
**Age**	
20-29	118/160 (73.8)
30-39	239/328 (72.9)
40-49	114/150 [76]
50-59	19/36 (52.8)
60 and over	1/2 [50]
**Vaccine hepatitis B**	
Not vaccinated	137/180 (76.1)
Vaccinated	354/496 (72.4)

In the 491 staff, a total of 780 injuries were reported. Overall, hollow-bore needles were responsible for 361 (46.3%) of all sharps injuries, while non hollow-bore needle and glass were responsible for 206 (26.4%) and 149 (19.1%) of the injuries. Only 64 (8.2%) injuries were due to solid objects.

Re-capping a needle was the first cause of the injury (24.5% of all injuries) reported by health care workers. Moreover, 13.2% of needlestick and sharps injury of health care workers were self-inflicted, and 12.4% of injuries were inflicted by their colleagues during surgical interventions. In addition, 12.8% of injuries were due to suturing by a surgical suture needle.

Views and knowledge assessment is summarised in table 2. In terms of views, staff appeared satisfied with employers safety measures (92.5%); although most wanted all patients to be tested for HIV (89.2%).

**Table 2:** Assessment of views and knowledge Percentage of health staff that agree with the following statements.

Views		Agree	
"My employer provides adequate safety measures to minimize HIV and viral hepatitis transmission"		613	92.5%
"I have the right to be informed if an HIV positive patient or a patient with viral hepatitis is present in my direct work area"		581	88.8%
"All patients admitted to hospital or attending hospital clinics should be tested for HIV"		589	89.2%

**Knowledge**	**Correct answer**	**Answering correctly**	

"Universal precautions are applied to patients with HIV and viral hepatitis only"	False	459	32.1%
"Isolation is necessary for patients with blood-borne infections"	False	473	30.0%
"Used needles can be recapped after giving an injection"	False	555	17.9%
"For decontamination of devices (with only contact with skin) washing with usual detergent is enough"	Correct	275	40.7%
"Universal precautions are not necessary in situations that might lead to contact with saliva"	Correct	192	28.4%
"HCWs with non intact skin should not be involved in direct patient care until the condition resolves "	Correct	459	67.9%
"Blood spills should be cleaned up promptly with sodium hypochlorite"	Correct	549	81.2%

In terms of knowledge, responses were poor, with the correct response rate under 50% for 5 or the 7 questions: most thought universal precautions were for HIV and hepatitis only, that isolation was necessary, and used needles could be re-capped. The only two questions with a correct response rate of more than 50% were in relation to patients with "non-intact skin" should avoid direct patient care, and "blood spills should be cleaned up promptly with sodium hypochlorite".

## Discussion

Reported needlestick and sharps exposure in health care staff in Kabul is high, mainly from hollow-bore needle injuries, particularly during re-capping. The result is similar to the findings of studies elsewhere [8,12-17]. International data show that the hollow-bore needle, due to its nature of containing the residual blood and other fluids, is the most hazardous instrument among medical sharps devices. However, avoiding re-capping used needles and minimizing the unnecessary use of needle has had remarkable success in the reduction of needle injuries in other countries [18].

The study showed a low level of knowledge of the basic principles of universal precautions. Many understood that universal precautions should not be applied to patients with HIV and viral hepatitis only, the majority of them stated that isolation is necessary for patients with blood-borne infections, and that needles can be re-capped after an injection.

Implementing a training package may help, but systems need to be in place as well. Universal precautions provide protection from a range of blood-borne pathogens, but their effectiveness relies upon the knowledge of health care workers and the level of compliance in their use [19].

## Conclusions

Bearing in mind that universal precautions play an important role in minimising and preventing exposure of health care workers to pathogens [9] there is a need for developing strategies to promote the use of universal precautions which take into account behaviour change and accrual of knowledge including its integration into practice. The country needs an obligatory training programme in universal precautions for health staff, involvement of senior health staff in the policies and their implementation, and systems for monitoring the appropriate use of equipment, and establishing post exposure reporting system. Finally, routine immunisation of health care workers against hepatitis B is required. The Ministry of Public Health should seek an appropriate mechanism to vaccinate all health care workers throughout the country.

As this study was conducted in Kabul public hospitals, similar studies are required to document the pattern of sharps injuries and universal precautions throughout the country, although it is unlikely to be any better outside Kabul.

## Competing interests

The authors declare that they have no competing interests.

## Authors' contributions

Ahmad Shah Salehi contributed in the design, data collection and statistical analysis of the study as well as the draft of the manuscript. Paul Garner contributed in the design, revision and re-drafting of the manuscript. Both authors read and approved the final manuscript to be published.

## Pre-publication history

The pre-publication history for this paper can be accessed here:

http://www.biomedcentral.com/1471-2334/10/19/prepub
